# Hydrops fetalis and neonatal abdominal compartment syndrome continuum from immature gastric teratoma: a case report

**DOI:** 10.1186/s12887-020-02090-0

**Published:** 2020-04-27

**Authors:** Alvin B. Caballes, Leona Bettina P. Dungca, Maria Esterlita V. Uy, Maria Geraldine C. Torralba, Cristina Marie G. Embuscado

**Affiliations:** 1 College of Medicine, University of the Philippines Manila, Manila, Philippines; 2 Philippine General Hospital, Manila, Philippines

**Keywords:** Abdominal compartment syndrome, Hydrops fetalis, Teratoma, Case report

## Abstract

**Background:**

Hydrops fetalis as well as abdominal compartment syndrome (ACS) are conditions that are associated with high mortality rates. A rare case of immature gastric teratoma causing fetal hydrops and subsequent ACS is presented. The related pathophysiologic mechanisms are discussed, and the importance of timely recognition and appropriate interventions are highlighted.

**Case presentation:**

The male patient was born preterm, weighing 3.9 kg., by Cesarean section. Prior prenatal ultrasounds were normal, but a scan done just before delivery had findings indicating polyhydramnios, fetal ascites, and meconium peritonitis. Upon delivery, the patient had respiratory distress, anasarca and a massively distended abdomen. Resuscitation measures, including ventilatory support, were instituted. Imaging studies showed ascites as well as a large, complex intra-abdominal lesion with calcifications. In the succeeding hours, anuria persisted, anasarca worsened, the abdomen became more distended, and inotrope requirements increased. The occurrence of ACS, from what was presumed to be a retroperitoneal teratoma, was therefore considered. Laparotomy was done on the 28th hour of life, with en bloc excision of a massive tumor and attached section of the greater curvature of the stomach. Passage of urine occurred intra-operatively, and the patient was soon after weaned off inotropes and ventilator support. The histopathologic result was immature gastric teratoma. No chemotherapy was given, and the patient’s serum AFP is at normal levels 15 months following surgery.

**Conclusion:**

The presence of a massive intra-abdominal lesion can result in the pathophysiologic continuum of hydrops fetalis and neonatal ACS. The early recognition of such an association can enable appropriate expectant management of similarly affected neonates, including emergent decompression laparotomy.

## Background

Non-immune hydrops fetalis has varied etiologies, including abdominal tumors. Despite proper diagnoses and therapies, mortality rates for affected fetuses and newborns are still considerable [1]. The persistence of the underlying cause beyond the fetal period may result in additional adverse consequences. Such a situation is demonstrated in the presented case, where an abdominal tumor led to the development of hydrops fetalis and subsequent neonatal abdominal compartment syndrome (ACS). The postulated pathophysiologic relationships of these conditions are discussed.

## Case presentation

**Fig. 1:**
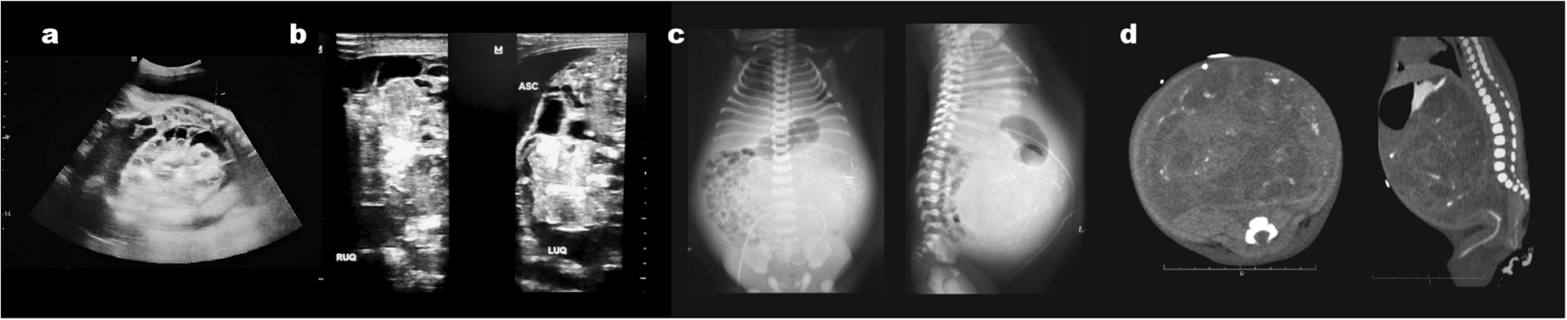
Imaging studies: **a**. Prenatal ultrasound showing fetal ascites and matted bowels with scattered calcifications; **b**. X-ray AP and lateral views indicating an intra-abdominal soft tissue density with calcifications, elevating the diaphragms and displacing the bowels to the right and posteriorly; **c**. Ultrasound images showing a large, complex abdominopelvic focus (9.4 cm in its widest dimension) with calcifications and moderate ascites (in the perihepatic, perisplenic and pelvic areas); **d**. CT transverse and sagittal views showing an intra-abdominal complex focus with internal septations and calcifications (measuring 10.1 × 13.2 × 8.2 cm), displaced viscerae, and moderate ascites

The male patient was born preterm, at 36 and 5/7 weeks of gestation, to a 26 year old primigravid mother. The latter, known to have an intracranial vascular anomaly, had received prenatal care at another hospital. Five prior prenatal ultrasound studies apparently had normal results. However, an ultrasound scan done two days before delivery showed polyhydramnios, fetal ascites and meconium peritonitis. A repeat study was done the following day by another obstetric sonologist, and had similar findings. An impression of meconium peritonitis was made supposedly based on the documentation of dilated and aperistaltic bowel loops with thickened walls and areas of calcification (Fig. 1a). The estimated fetal weight was 5.16 kg. The mother was referred to the authors’ institution, in anticipation of the need for dedicated neonatal surgical and intensive care services. As the mother was in labor upon arrival at the receiving hospital, and considering the expected dystocia as well as heightened maternal risk from an intracranial lesion, emergency Cesarean section was done.

Upon delivery, the patient was noted to be in respiratory distress, anasarcous, and had a markedly distended abdomen. The 1-min APGAR score was at 5. Endotracheal intubation and bag ventilation were immediately done. With the ensuing APGAR score improved at 8, the patient was transferred to the neonatal intensive care unit. The patient was hooked to a mechanical ventilator, IMV mode, with the following settings: FiO2 100%, RR 60/min, PIP 20 cmH2O, PIP 5 cmH2O, Inspiration time 0.4 s. Nasogastric and urethral catheters were inserted and the umbilical vein was cannulated. Dopamine and Dobutamine drips, both at 10 μg/kg/hour, were started. Pediatric aging was at 35 weeks. The newborn weighed 3.9 kg, with a body length of 48 cm. **(z-score − 0.52)**, and head, chest and abdominal circumferences were 34 cm **(z-score 0.10)**, 36 cm, and 45 cm, respectively. The patient and mother had the same blood type, B+. Ampicillin was started, while Amikacin was put on hold due to the patient’s uncertain renal status, as discussed subsequently.

The baseline x-ray films showed a soft tissue density with scattered calcifications predominantly occupying the left hemi-abdomen (Fig. 1b). An ultrasound study demonstrated moderate ascites and a large, complex abdominopelvic focus with calcifications (Fig. 1c). The latter was interpreted to be matted bowels possibly due to meconium pseudocyst formation. Neither kidney could, however, be definitively delineated. As the patient was also anuric, the likelihood that the lesion either primarily involved the kidneys, as with a mesoblastic nephroma, or had otherwise markedly displaced, compressed or even invaded these, ostensibly from a massive neuroblastoma or a retroperitoneal teratoma was entertained. Hence, a CT scan was done to better define the abdominal anatomy. The plain study demonstrated an intra-abdominal complex focus with septations and calcifications, ascites, and, importantly, the presence of both kidneys (Fig. 1d). The radiologists’ primary impression was meconium peritonitis. A patent ductus arteriosus with moderate tricuspid regurgitation, mild pulmonary regurgitation, and severe pulmonary artery hypertension were documented on 2D echo.

In the meantime, the patient’s anasarca and abdominal distention became more pronounced. The patient’s hemodynamic status was labile, and epinephrine and norepinephrine drips, initially at 0.05 μg/kg/min, were started for additional inotropic support. The repeat blood gas study showed persistent hypoxemia (PaO2 50.9 mmHg) and respiratory acidosis (pH 7.29, PaCO2 42 mmHg). The platelet count fell from a baseline of 304 to 21.3 (X10^9/L), and serum creatinine was at 121 mg/dl. The clinical and laboratory parameters were thus indicative of end-organ dysfunctions [2]. The earlier consideration for a renal pathology drew attention to detecting urine flow from the indwelling catheter, such that the latter was not utilized for measuring abdominal pressure. Still, a reassessment of the patient’s clinical course and work-up results prompted the recognition of ACS from a primary abdominal lesion, presumably a retroperitoneal teratoma. With no effective relief attainable from medical interventions, the need for decompression laparatomy was raised. The parents were appraised of the patient’s condition as well the indication for and risks of the procedure. After an informed consent was obtained, the patient was promptly brought to the operating room. Surgery commenced on the 28th hour of life.

Due to the patient’s already precarious condition, it was felt by the surgeons and anesthesiologists that laparotomy had to be done expeditiously - and attempting to secure additional vascular access beforehand, particularly with patient’s markedly edematous state, would only cause undue delay. Thus, after induction of anesthesia and preparation of the operative site, the umbilicus was cored out, with the cannulated vein maintained as the sole vascular access site. A transverse supra-umbilical laparotomy was made, sparing the falciform ligament and attached umbilical vein. A short vertical midline incision was subsequently done from the laparotomy site towards the already detached umbilicus, enabling the latter, together with the cannulated vein, to be moved and anchored temporarily at the cephalad edge of the laparotomy wound. The tumor was thereafter freed from the adjacent structures, using electrocautery and sharp dissection, and gradually delivered out of the wound. The procedure was repeatedly paused due to episodes of hypotension and bradycardia, which required boluses of crystalloids and blood products. The tumor was eventually excised en-bloc with a rim of the greater curvature of the stomach. The gastric defect was repaired with two layers of sutures, and the umbilicus was reattached upon closure of the abdominal wound. Ventilatory compliance improved and urine passage was noted as the abdomen was decompressed during surgery. The procedure lasted 2 h, and 160 cc of packed rbc, aside from other blood components, was transfused. The resected tumor weighed 570 g.

Post-operatively, the patient was rapidly weaned off mechanical ventilation as well as inotropic support. In the succeeding days, the umbilical catheter was removed and peripheral vascular access established. Partial parenteral nutrition, earlier provided at 60 kcal/kg/day, was discontinued and enteral feedings were gradually provided by orogastric tube. Sepsis as well as gastroesophageal reflux were intervening problems which were managed accordingly. By the thirtieth day, the patient was already feeding well and had no other medical problems. He was discharged on the thirty-second day, with a body weight of 2.5 kg **(z-score − 3.69)** and abdominal circumference of 34 cm. The histopathologic interpretation for the excised tumor was immature teratoma, Grade 3, with 40% immature neural epithelium. The initial serum AFP level was 2879 ng/ml, which was within acceptable newborn limits. The patient had monthly out-patient check-ups, inclusive of serum AFP determinations. No chemotherapy was administered. He is well and thriving, 15 months after surgery, with the latest AFP at 3.9 ng/ml. The patient’s parents have been very satisfied with the care and outcome of the patient and have given their consent for this report.

## Discussion and conclusion

The initial primary diagnosis for the patient, that of meconium pseudocyst, was based primarily on earlier interpretations of sonographic and X-ray studies. As already pointed out, the persistence of anuria and the non-delineation of the kidneys by ultrasound subsequently pointed to the possibility of primary renal anomalies, or tumor encroachment. Nonetheless, the massive lesion, with its heterogenous and calcific content and distinct border, as well as its extensive involvement of the retroperitoneal area, as documented by CT scan, made the surgeons more inclined to consider a retroperitoneal teratoma. That the identified kidneys were compressed along the anteroposterior axis, yet not significantly displaced, made neuroblastoma, though a plausible differential, a less likely possibility.

Teratomas are the most frequently encountered type of germ cell tumor in neonates and infants [3]. However, retroperitoneal as well as gastric teratomas are rare. In prenatally diagnosed fetuses, teratomas have been found, in decreasing frequency, in the following areas: sacrococcygeal (61%), cervical (20%), mediastinal (9%), intracranial (3%), nasopharyngeal (3%), and retroperitoneal (1%) [4]. Across all age groups, only 1% of teratomas arise from the stomach and predominantly affect male patients of Asian descent [5]. A palpable mass or abdominal distention are the most common presenting signs, followed by mass effects, such as respiratory distress or features of bowel obstruction. Infrequently, gastrointestinal bleeding can occur for patients with intra-luminal lesions. In utero gastric obstruction is conjectured to cause polyhydramnios. Likewise, massive tumors can lead to dystocia, a fetal indication for Cesarean delivery, as with the current case. While most gastric teratomas are located at the greater curvature, these have also been found in other areas of the stomach and can even involve the entire organ [6]. The associated ultrasound and radiologic findings, that of a large heterogenous lesion with calcifications, are non-specific, however. Thus, as exemplified in the presented case, other more frequently encountered entities, especially in the newborn period, are more likely to be considered. The diagnosis is therefore mostly arrived at only during surgery and definitively confirmed on histopathologic examination. Similar to other teratomas, these tumors are histologically classified as either mature or immature. About 25% of gastric teratomas are histologically immature, and these are found mostly in neonates or young infants [5–11]. The presence of malignant germ cell elements, which can be overlooked in immature tumors, signify other diagnoses, principally yolk sac tumor. Embryonal and yolk sac-derived tumors synthesize AFP, and the initial serum value may relate to the presence of these malignancies, while sequential determinations after removal of the tumor are useful for detecting recurrence [4]. As the actual relationship between histologic grading and malignancy cannot be definitively substantiated, chemotherapy has anecdotally been given to patients with high grade as well as recurrent tumors [5, 8, 11]. For the current case, chemotherapy was not given, and continued surveillance will be done, as the risk of recurrence remains.

The patient was also diagnosed as having hydrops fetalis. By definition, at least two areas of pathologic fluid accumulation should be present to qualify for the diagnosis. The current patient had ascites as documented on prenatal ultrasound with anasarca found only at the time of delivery. As there was no set-up for red cell alloimunization, the patient’s hydrops was of the non-immune type. The prognosis for patients in the latter group depends on several factors, including the underlying cause, age at detection, age at delivery, and immediate neonatal status, among others. Mortality rates for patients with non-immune hydrops fetalis can be as high as 50–60% [1].

Several abdominal and pelvic conditions have been reported to be associated with the development of hydrops fetalis [12]. Though the authors were not able to retrieve any prior report of hydrops fetalis occurring in a patient with immature gastric teratoma, the condition has been documented to be associated with the differential diagnoses considered in this case - meconium peritonitis, mesoblastic nephroma, and neuroblastoma [12–14]. Among teratomas, hydrops fetalis has been most commonly reported among patients with sacrococcygeal tumors [4, 12, 15]. It is postulated that hydrops results from cardiac failure, which, in turn, may be due to vascular shunting, decreased venous return, anemia, or other hemodynamic abnormalities that may be brought about by the underlying fetal condition [12, 15, 16]. Similar mechanisms may have led to the occurrence of hydrops fetalis in the present case, even as such became apparent only in late in gestation. The relatively late detection of the tumor suggests a delayed but rapid growth of the primary lesion, as has also been ultrasonically documented in another neonatal gastric teratoma case [10]. The sudden expansion of the lesion would have led to rapidly increased tumor blood flow or intra-abdominal pressures, either of which could have predisposed to the development of non-immune hydrops fetalis. Unfortunately, as no fetal Doppler studies were done, the actual hemodynamic changes for the presented case was not ascertained. The ex-utero transformation facilitated other adverse developments. With the transition from placental to pulmonary gas exchange, the consequences of the restricted ventilatory capacities became apparent soon after birth. Neonatal resuscitation would have also led to reperfusion injury and its consequent inflammatory response. Such would have aggravated body fluid shifts, which would then have further increased intra-abdominal pressures [17].

Elevated intra-abdominal pressure, or intra-abdominal hypertension (IAH) can occur in several clinical scenarios - diminished abdominal wall compliance, increased intra-abdominal or intra-luminal contents, interstitial fluid accumulation, and others [17]. The increased abdominal pressure can lead to local effects, such as visceral hypoperfusion and its consequences (e.g., the anuria observed in the present case), as well as more systemic sequela, including further tissue damage brought about by hypoxemia from impaired ventilation [18]. At a critical pressure threshold, ACS ensues, wherein vital organ functions become compromised. For adults, IAH is defined as the sustained or repeated pathological elevation in IAP ≥ 12 mmHg, while ACS is the sustained IAP ≥ 20 mmHg that is associated with the onset of organ dysfunction. The cut-off values are lower for children for both measures, at 10 mmHg [19]. The monitoring of intra-vesical pressures (IVP) as a proxy measure of intra-abdominal pressures has been recommended [17, 19]. Nonetheless, there are limitations with this approach, and thresholds are yet to be set for neonates [20]. The IVP was not measured in the present case. Still, it has also been proposed that, in critically ill neonates with tense abdomens, the presence of clinical and diagnostic indicators of end-organ dysfunction can be sufficient to diagnose ACS and intervene accordingly [20]. The therapeutic options include measures to improve abdominal wall compliance, optimize fluid management, drain intraluminal contents, remove space-occupying lesions, and provide support for compromised organ systems [21]. The value of urgent decompression laparotomy for severe or recalcitrant ACS, which can also include the excision of the offending intra-abdominal lesion as with the current case, cannot be overemphasized [22].

ACS, while long being a concern particularly for pediatric surgeons (specifically in managing abdominal wall defect cases), reportedly remains under-recognized and therefore under-treated in the pediatric population [23, 24]. The collective experience with immature gastric teratoma epitomizes this situation. Recently reported cases of neonates with immature gastric teratomas also presented with massive tumors as well as respiratory distress [5–11]. While the concurrent presence of ACS in these cases may be surmised, such a consideration was not distinctly stated in the reports. All the cited cases also underwent emergent excisions. However, one patient succumbed to sepsis and another subsequently manifested with hypoxic brain injury [6, 7]. ACS-related tissue hypoperfusion may have led to these complications, possibly even causing intestinal ischemia, bacterial translocation and subsequent sepsis in the former case. The same circumstances could have contributed to the post-operative septic course for the present patient.

**Fig. 2:**
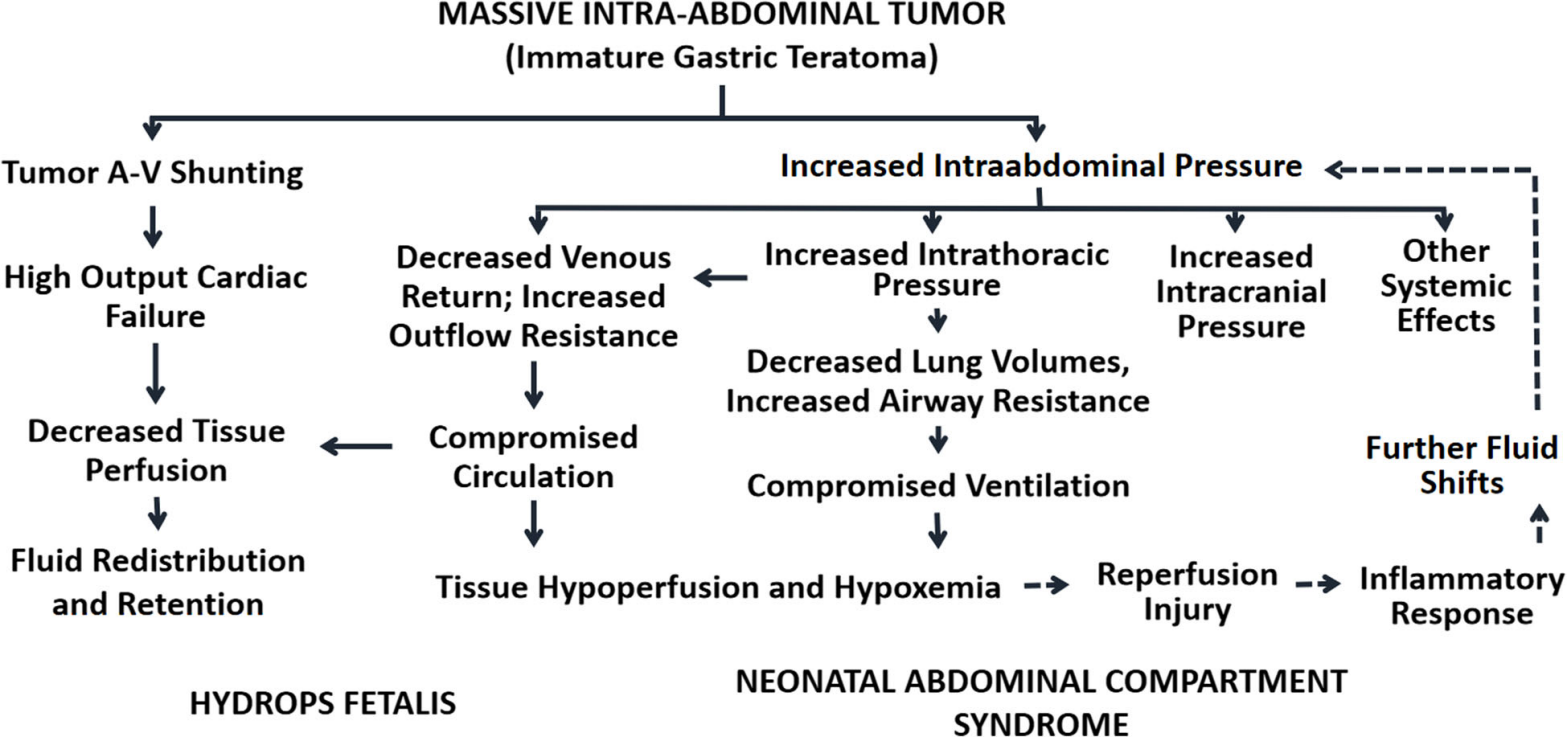
Postulated inter-related pathophysiologic mechanisms for the development of hydrops fetalis and neonatal abdominal compartment syndrome in a patient with massive congenital intra-abdominal tumor

The presence of an immature gastric teratoma and the resultant hydrops fetalis, and neonatal ACS are inter-related conditions, for which a pathophysiologic continuum is surmised (Fig. 2). The further implication would be that the early recognition of such a relationship may enable timely interventions in similar cases. Needless to say, even early and aggressive treatments cannot guarantee uniformly successful outcomes for these patients [25].

In summary, the report highlighted the clinical features, course, and management of a case of gastric teratoma associated with hydrops fetalis and neonatal ACS. The characterization, differential diagnoses, and management approaches for these conditions were discussed. The underlying relationships, by way of a pathophysiologic continuum, was postulated. The early recognition of such an association can enable appropriate expectant management, including emergent decompression laparotomy, of similarly affected neonates.

## Data Availability

Data sharing is not applicable to this article as no datasets were generated or analysed during the current study.

## Abbreviations

ACS: Abdominal compartment syndrome
AFP: Alpha fetoprotein
IAH: Intra-Abdominal hypertension
IVP: Intra-vesical pressure
